# Two Novel Bacteriophages Control Multidrug- and Methicillin-Resistant *Staphylococcus pseudintermedius* Biofilm

**DOI:** 10.3389/fmed.2021.524059

**Published:** 2021-03-31

**Authors:** Sang Guen Kim, Sib Sankar Giri, Saekil Yun, Sang Wha Kim, Se Jin Han, Jun Kwon, Woo Teak Oh, Sung Bin Lee, Yong Ho Park, Se Chang Park

**Affiliations:** ^1^Laboratory of Aquatic Biomedicine, College of Veterinary Medicine and Research Institute for Veterinary Science, Seoul National University, Seoul, South Korea; ^2^Department of Veterinary Microbiology, College of Veterinary Medicine, BK21 Plus Program for Veterinary Science and Research Institute for Veterinary Science, Seoul National University, Seoul, South Korea

**Keywords:** *Staphylococcus pseudintermedius*, antibiotic resistance, bacteriophage, biofilm, methicillin-resistance, methicillin-resistant *Staphylococcus pseudintermedius*

## Abstract

As a primary bacterial pathogen in companion animals, *Staphylococcus pseudintermedius* has zoonotic potential. This pathogen exhibits multidrug resistance, including resistance to methicillin, and biofilm-forming ability, making it hard to eradicate with antimicrobial agents. One potential alternative is bacteriophage therapy. In this study, we first characterized the antimicrobial resistance profile of *S. pseudintermedius* from canine samples and isolated two novel bacteriophages, pSp-J and pSp-S, from canine pet parks in South Korea to potentially control *S. pseudintermedius*. The biological characteristics of phages were assessed, and the phages could infect most of the methicillin-resistant *S. pseudintermedius* strains. We found that these phages were stable under the typical environment of the body (~37°C, pH 7). We also assessed bacterial lysis kinetics using the two phages and their cocktail, and found that the phages could prevent biofilm formation at low doses and could degrade biofilm at high doses. Taken together, this study demonstrates that bacteriophages pSp-J and pSp-S isolated in this study can be used to potentially treat methicillin-resistant *S. pseudintermedius*.

## Introduction

*Staphylococcus pseudintermedius* is an opportunistic pathogen commonly isolated from dogs and other companion animals. Given the recent rise in pet ownership, the number of reported cases of *S. pseudintermedius* infection in humans has risen as well (1, 2). Moreover, with this increased incidence rate, methicillin-resistant *S. pseudintermedius* (MRSP) has emerged, thereby raising concerns for both veterinary and human health (3–5).

MRSP can adhere to medical devices, such as implants and suture materials, and form a biofilm, which makes treatment challenging (6, 7). A biofilm is an aggregate of the planktonic (freely floating) bacterial cells enclosed in a matrix of self-produced materials, including nucleotides, proteins, and exopolysaccharides that can adhere to biotic or abiotic surfaces (8, 9). Compared to planktonic cells, bacterial cells in biofilms can change their mode of metabolism, have enhanced cell to cell communication, and are highly tolerant of harsh environmental conditions, such as the presence of antibiotics since the matrix blocks external stresses (10). Because the matrix provides competitive advantages for survival and colonization, staphylococci in biofilms can tolerate more than 100-fold concentration on the antibiotics (11). Therefore, there is an urgent need for strategies to control antibiotic resistance and biofilm formation in the event of bacterial infection.

One potential alternative is bacteriophage therapy. Bacteriophages (phages) specifically infect bacterial hosts and inhibit host growth. Lytic phages are major candidates as alternatives to antibiotics since they spread only by lysing the host bacterial cells. Theoretically, even just a single particle of a phage can degrade biofilms because its progeny will infect adjacent bacterial cells. Recently, phages have shown the ability to degrade the extracellular matrix of *Staphylococcus* biofilms (12–14). Some of them are already available in commercialized solutions (15–17). A number of trials have verified the safety of phage application in both animal models and the clinical setting (18–20). Some clinically effective phages have already been designated in the generally recognized as safe (GRAS) list of the Food and Drug Administration of the USA as food additives, suggesting that phages are generally safe for human use. The wild-type phages (not genetically modified) are inherently present in the nature and already close to our life even included in food supply and may therefore be isolated from different sources (21).

In this study, we first assessed the prevalence of antimicrobial-resistant *S. pseudintermedius* in South Korea. And then, we isolated two novel phages, pSp-J and pSp-S, from soil and water samples from pet parks in South Korea for possible application in controlling MRSP. We characterized these phages and assessed their stability in different environmental conditions. We also determined their anti-bacterial and anti-biofilm activities. We found that these phages, pSp-J and pSp-S, can prevent biofilm formation at low doses and degrade biofilm at high doses, suggesting that these phages may potentially be used for treatment of MRSP infection.

## Materials and Methods

### Ethical Statement

The procedures involving animals were carried out under the informed consent of the owner. To ensure the well-being of involved animals, the procedures were carefully performed by a veterinarian. The animal protocols were reviewed and approved by the Institutional Animal Care and Use Committee (IACUC) of Seoul National University (SNU-180718-3).

### Bacterial Isolation and Culture Conditions

Samples were swabbed from the external auditory meatus, anus, skin, and oral cavity of 208 infected dogs that visited the local veterinary hospitals in Seoul and Gyeonggi-do Korea in 2018 and 2019. The swabs were streaked on sheep blood agar (MB Cell, LA, CA) and incubated at 37°C for 1 day. Based on morphology, the presumptive staphylococci-like colonies were selected and identified using matrix-assisted laser desorption ionization-time of flight (MALDI-TOF) (22). The identification was confirmed by polymerase chain reaction (PCR) using a set of *S. pseudintermedius* species specific primers (23). *Staphylococcus* strains were cultured in tryptic soy broth (TSB; Becton Dickinson, Franklin Lakes, NJ, USA) with shaking or sub-cultured on tryptic soy agar (Becton Dickinson) at 37°C. All bacterial strains used in this study are listed in Table 1.

**Table 1:** Host range of phage pSp-J and pSp-S against *S. pseudintermedius* strains used in this study.

**MR^a^ status**	**Strain**	**EOP^b^ (plaque clarity^c^)**	**Source**	
		**pSp-J**	**pSp-S**	
MR	1D1	0.009 (C)	0.007 (T)	(22)
	1D1V	0.005 (C)	0.011 (C)	
	3H1-2^d^	0.006 (C)	0.011(C)	
	3H1-2V^d^	0.0008 (T)	0.015 (C)	
	4D1	0.17 (C)	0.09 (C)	
	4D1V	0.08 (C)	0.06 (C)	
	5D1	0.0005 (T)	0.013 (C)	
	5D1V	0 (N)	0.05 (C)	
	7D1	0.5 (C)	0.4 (C)	
	8D1V	0.07 (C)	0.06 (C)	
	8H1-10^d^	0.28 (C)	0.2 (C)	
	9D1	0 (N)	0.07 (C)	
	9D1V	0 (N)	0.002 (T)	
	32-2	0.009 (C)	0.022 (C)	
	47-5	0.008 (C)	0.015 (C)	
	54-1^d^	0.06 (T)	0.04 (C)	
	A-ISB^d^	0 (N)	0.08 (C)	
	C21-2-1^e^	0.37 (C)	0.55 (C)	This study
	C27-6	0.04 (T)	0.005 (T)	
	C28-6-1	0 (N)	0 (N)	
	C28-6-2	0.005 (T)	0 (N)	
	C30-4-1	0.4 (T)	0.6 (C)	
	C40-4-1	0.05 (C)	0.02 (C)	
	C41-4	0 (N)	0.011 (C)	
	C49-1	0.017 (T)	0.021 (C)	
	C55-4-1	0.44 (C)	0.005 (T)	
	J37-2	0.7 (C)	0.4 (C)	
	Kw34-4-2	0.5 (C)	0.5 (C)	
	Kw34-3	0.26 (C)	0.35 (C)	
	Kw34-5-2	0 (N)	0 (N)	
	Kw28-1	0.4 (C)	0.6 (C)	
	Kw28-5	0 (N)	0 (N)	
	Kw33-3-2	0 (N)	0.005 (T)	
	Kw34-2	0.08 (C)	0.23 (T)	
	In36-4	0 (N)	0.05 (C)	
	In37-4-1	0.0017 (T)	0.001 (T)	
	In47-4-1	0.002 (T)	0.003 (T)	
	In47-4-3	0 (N)	0.006 (T)	
	In48-4-2	0.001 (T)	0.0001 (T)	
	In50-4-1	0.001 (T)	0.0002(T)	
MS	6D1	0.0022 (T)	0.014 (C)	[22]
	6D1V	0.004 (T)	0.011 (C)	
	8D1	0.0022 (C)	0.0015 (C)	
	8H1-10V^d^	0.29 (C)	0.09 (C)	
	D10-3	0.11 (C)	0.09 (C)	
	D13-1	0.006 (C)	0.011 (C)	
	D20	0.44 (C)	0.08 (C)	
	A-D4-1	0.004 (T)	0.017 (C)	
	Kw31-4-3	0.09 (C)	0 (N)	This study
	Kw41-4-1	0.008 (T)	0 (N)	
	In41-4-2	0.002 (T)	0.004 (T)	
	In42-4-2	0.001 (T)	0.003 (C)	
	In55-4-3	0 (N)	0.014 (T)	

a *MR, methicillin-resistance; MS, methicillin-susceptible*.

b *EOP, efficiency of plating; the results of a spot assay using 10-fold serial dilutions of phage lysate*.

c *C, clear plaque; T, turbid plaque; N, no plaque*.

d *Strains isolated from veterinary staff*.

e *Indicator strain*.

### Antimicrobial Suceptibility

The antimicrobial susceptibility profiles of the isolates were determined with disk diffusion method in accordance with the Clinical and Laboratory Standards Institute (CLSI) guidelines [24]. Antimicrobial agents that are generally used in veterinary hospitals in Korea were purchased from Oxoid and tested: penicillin (10 U), oxacillin (30 μg), cefazolin (30 μg), cefoxitin (30 μg), cefotaxime (30 μg), vancomycin (30 μg), gentamicin (10 μg), amikacin (30 μg), quinupristin-dalfopristin (15 μg), rifampin (5 μg), chloramphenicol (30 μg), trimethoprim-sulfamethoxazole (1.25/23.75 μg), ciprofloxacin (5 μg), minocycline (30 μg), tetracycline (30 μg), linezolid (30 μg), and erythromycin (15 μg). Shortly, an overnight culture of *S. pseudintermedius* was prepared in Muller–Hinton broth (Becton Dickinson) and was added to sodium chloride solution (0.45%; w/v) to achieve 0.5 McFarland standard turbidity. Then, the bacterial solution was inoculated by spread plating on Muller–Hinton agar using a sterile cotton swab. After allowing the inoculum to dry, the discs containing antimicrobials were placed onto the agar, and the cultures were incubated for 18–24 h at 37°C. A reference strain, *Staphylococcus aureus* (ATCC 25923), was used as control.

### Phage Isolation, Purification, and Propagation

For isolation of phages that infect *S. pseudintermedius*, we collected 150 water samples and 50 soil samples all over the South Korea for 5 months and used the MRSP strain (C21-2-1) to screen for the phages. Previously grown host strain was inoculated into a 1:1 mixture of the collected sample and TSB, followed by incubation for 24 h at 37°C. After enrichment, 10 μL of 10-fold serial dilutions (10^−1^ to 10^−8^) of culture broth were spotted on the bottom agar layered with the host strain. Samples that resulted in inhibition zones were collected by centrifugation and membrane filtration to confirm the presence of plaques using the double-layer agar method. After overnight incubation at 37°C, the plaque was cloned five times by sub-culturing a single plaque. Two phages with small and clear plaque were isolated from the different samples, designated pSp_SNUABM-J (pSp-J) and pSp_SNUABM-S (pSp-S), and used for further study.

### Electron Microscopy

For transmission electron microscopy analysis, the phages were precipitated with polyethylene glycol-NaCl and re-suspended in SM buffer (100 mM NaCl, 50 mM Tris pH 7.5, and 10 mM MgSO_4_). Then, 10 μL of the phage solution (>10^10^ PFU/mL) was loaded on a copper grid, and the excess solution was removed using a filter paper. The phages were stained with 2% uranyl acetate for 1 min and washed three times with distilled water. The grid was air-dried, and the image was obtained with a Talos L120C transmission electron microscope (FEI, OR, USA) operated at 120 kV. The dimensions of the phages were calculated by measuring five independent virions.

### Host Range Analysis

The host ranges of pSp-J and pSp-S were determined by spot assay against 54 strains of *S. pseudintermedius* (6 and 48 strains from veterinary staff and canine, respectively), including the 29 strains isolated in this study and 25 strains reported in a previous study (**Table 1)** (22). A drop of phage solution (>10^7^ PFU/mL) was inoculated into TSA plate overlaid with each bacterial strain mixed with top agar. The plates were incubated for 18–24 h at 37°C, and the lytic ability of the phages were evaluated by the clarity of the lysis zone, which was assessed as: clear (C), turbid (T), or no lysis (N; Table 1). The efficiency of plating (EOP) of the phages was assessed, which was found to be similar to that of spot assay. To avoid the lysis from without, a high concentration of phage solution was not used for the spot assay. Ten microliters of the 10-fold serial dilutions (10^3^-10^7^ PFU/mL) was spotted on the host strain-overlaid TSA plate. The EOP values were calculated as the ratio of the average PFUs of a susceptible strain to the reference strain (C21-2-1) with three replicates.

### Adsorption Assay and One-Step Growth Curve

The adsorption assay was carried out as previously described (24). Briefly, the host strain at the exponential growth phase (2 × 10^8^ CFU/mL) was infected with the phage solution at an MOI of 0.001. Samples were taken at 0, 1, 3, 5, 10, 15, and 20 min post-infection and promptly diluted in 0.1% peptone water, followed by centrifugation at 4°C. The concentrations of the free phages were determined using the supernatants. The growth curves of pSp-J and -S were constructed by inoculating the phage solution to host strain cultures at the log phase. The phages pSp-J and -S were allowed to adsorb to the host for 5 and 10 min, respectively, followed by centrifugation. The supernatant was replaced with preheated TSB and incubated with shaking (150 rpm). Aliquots were taken every 10 min for 60 min, and the concentration of the phages at each time point was determined by the double-layer agar method. The concentration measurements were performed with three replicates.

### Thermal and pH Stability Assay

For thermal stability tests, 1 mL phage suspension (1.3 × 10^7^ PFU/mL) was incubated at 4 (control), 25, 37, and 50°C. For pH stability tests, 10 μL phage suspension (1.3 × 10^9^ PFU/mL) was used to inoculate 990 μL universalbuffer (10 mM KH_2_PO_4_, 10 mM Na-citrate, and 10 mM H_3_BO_4_) adjusted to pH 3.0, 5.0, 7.0 (control), 9.0, and 11.0 with either 1 M NaOH or 1 M HCl (25). The tubes were then incubated at room temperature (RT). Then, the samples were collected every 6 h for 24 h, and the phage titers were calculated using 10-fold serial dilutions of the aliquots at each condition for each time point using the double agar overlay plaque assay and converted into % value taking the count of the control group as 100%. All tests were performed with three replicates.

### Planktonic Bacterial Cell Lysis Assay

To evaluate the bacterial lysis effect of pSp-J, pSp-S and the cocktail (a 1:1 mixture of pSp-J and -S), host-phage co-culture was performed as previously described (26). Briefly, one percent of an overnight culture of the host cell was inoculated into fresh TSB to obtain 10^8^ CFU/mL, and the phage solution was inoculated into the host at different concentrations (MOI of 0, 0.001, 0.01, 0.1, 1, 10, 100, and 1,000). The broth was cultured with shaking, and the optical density at 600 nm (OD600) was measured every 30 min for 20 h. All tests were performed with three replicates.

### Biofilm Prevention Assay

To evaluate the biofilm prevention efficacy of the two phages, the host bacterial strain was co-cultured with the phages in 96-well-polystyrene plates (Nunc, Denmark). First, 1% of an overnight bacterial culture (10^8^ CFU/mL) broth was inoculated into fresh broth containing 1% D-glucose and was supplemented with the stock phage solution at different concentrations (10^3^, 10^4^, 10^5^, 10^6^, 10^7^, 10^8^, and 10^9^ PFU/ml). Then, 200 μL of aliquots were distributed into each well of the 96-well-plate and cultured at 37°C for 24 h without shaking. The supernatant was removed from the well, and the well was washed two times with PBS for removal of the remaining planktonic bacterial cells. Quantification of total biomass and the viable bacterial cell count in the biofilms was performed using standard plate count as previously described (26). The total biomass of the biofilm was quantified with crystal violet solution. Briefly, the biofilm was stained with crystal violet solution (1%) for 15 min. The excess stain was discarded, and the wells were washed twice to remove all the residual crystal violet solution. The crystal violet in the biofilms was dissolved in an ethanol-acetone solution (80:20, v/v) and the OD was measured at 595 nm (*n* = 4). For the enumeration of viable bacterial cells in the biofilm, the biofilms formed at the bottom of the plate were scraped and re-suspended in PBS. The serial dilutions of the solution were then directly spread and cultured on the TSA at 37°C for 24 h.

### Biofilm Degradation Assay

To evaluate the biofilm degradation efficacy of two phages, 1% of an overnight bacterial culture (10^8^ CFU/mL) was inoculated into fresh broth containing 1% D-glucose and cultured on 96-well-polystyrene plates for 24 h (*n* = 4). The biofilms were washed to remove the residual planktonic bacterial cells, and the phage solution with different concentrations (10^3^, 10^4^, 10^5^, 10^6^, 10^7^, 10^8^, and 10^9^ PFU/ml) were treated for 24 h. Then, the supernatant was removed from the well and washed twice. The total biomass and viable cell count of the biofilm were quantified as mentioned in section Biofilm prevention assay.

### Confocal Laser Scanning Microscopy (CLSM)

The biofilms were visualized by confocal laser scanning microscopy (CLSM) using Syto 9 green fluorescent nucleic acid stain (Invitrogen, CA, USA) as previously described (12). The biofilms were cultured on coverslips (22 × 22 mm; Paul Marienfeld GmbH & Co. KG, Lauda-Königshofen, Germany) in 6-well-plates for 24 h at 37°C. Then, the supernatant was discarded and washed twice to remove planktonic cells. After staining with Syto 9, the biofilms were examined under the microscope (SP8 X, Leica, Germany) with excitation at 488 nm and emission at 495–547 nm.

### Phage DNA Sequencing and Bioinformatics Analysis

The DNA of phage pSp-J, and -S was extracted as described in the previous report (26). The phage solution was treated with 10U of DNase I and RNase A (TakaraBio, Japan) at 37°C for 3 h. The nucleases were inactivated by EDTA. Then, proteinase K was treated at 50°C for 6 h and inactivated by incubating at 95°C for 15 min. Purification of the phage DNA in the solution was performed with conventional phenol-chloroform extraction methods (27). The library of NGS was constructed using NEBNext Ultra II DNA Library Prep Kit for Illumina (New England Biolabs, USA) according to the manufacturer's protocol. The purified DNA of the two phages was sequenced using an Illumina platform (HiSeq2500) and the reads were trimmed and assembled on the CLC Genomic Workbench (v6.5.1) at Genotech (Daejeon, South Korea). Prediction and annotation of open reading frames (ORFs) were performed using GeneMarkS, Rapid Annotation using Subsystem Technology (RAST) server, and BLAST with nr database (28–30). The presence of tRNA, antimicrobial resistance-, or virulence-related genes was observed by tRNAscan-SE (v2.0), ResFinder (v3.2), and VirulenceFinder (v2.0), respectively (31–33). Then, the genome map of the phages was visualized in the DNA plotter (34). Genome comparison was performed using the tBlastX algorithm and visualized in the easyfig (v2.2.3) (35). The phylogenetic analysis was performed with the whole genome sequences of the phages and the tree was built in the Virus Classification and Tree Building Online Resource (VICTOR) under D_0_ formula which is recommended settings for nucleotide sequences of prokaryotic viruses (36). The distances of intergenome were employed to infer a balanced minimum evolution tree with branch support (100 pseudo-bootstrap replicates) in the FASTME algorithm which is including SPR post processing for the D_0_ formula. At the midpoint, the phylogeny tree was rooted and visualized with FigTree (37).

### Pseudolysogeny Assay

PCR was performed to examine the presence of phage DNA in the host bacteria using the re-grown cells from the cell lysis assay. The chromosomal and extrachromosomal DNA was isolated as a previous description (38). The primers targeting major capsid protein of the phages (F: 5′-AAATCAACGTCAGCCGAAGC-3′, R: 5′-TGGCACGTCTGCAAACTCTA-3′; target size: 378 bp) were used. For the positive control of bacterial DNA, universal primers for 16s rDNA (27F, and 1492R) were used.

### Nucleotide Sequence Accession Number

The complete genome sequences of pSp-J and pSp-S were deposited in the NCBI GenBank under the accession numbers MT423823.1 and MT423824.1, respectively.

### Statistical Analysis

Statistical analysis were conducted using one-way analysis of variance (ANOVA) with the Bonferroni test on the SigmaPlot software (ver. 12.0, Systat Software, Inc., IL, USA). The value of *P* < 0.05 was considered as statistically significant.

## Results

### Antimicrobial Susceptibility of *S. pseudintermedius* Isolates

A total of the 29 *S. pseudintermedius* strains were newly isolated and 24 strains were from a previous study (39). The antimicrobial susceptibility results are presented in Supplementary Table 1. Of the 54 isolates, 41 strains (75.9%) showed methicillin resistance, and all strains displayed multidrug resistance except one MSSP strain which was susceptible to all the antimicrobial agents used in this study. In both methicillin-resistant and -susceptible strains, resistance to 5–8 antimicrobial agents was commonly observed. While MRSP strains were commonly resistant to penicillin (94.1%), tetracycline (92.6%), and trimethoprim-sulfamethoxazole (85.3%), MSSP strains showed resistance to penicillin (84.6%), erythromycin (76.9%), and trimethoprim-sulfamethoxazole (76.9%). All MRSP and MSSP strains were susceptible to amikacin, quinupristin-dalfopristin, and linezolid. For the safety of the researcher, the C21-2-1 strain which it showed resistance to methicillin while susceptible to many other drugs was chosen for subsequent screening of phages.

### Host Range of Bacteriophage pSp-J and pSp-S

Soil and water samples from pet parks in South Korea were used to isolate phages that infect *S*. *pseudintermedius*. Two phages were isolated from Jinju and Seoul, South Korea and designated as pSp-J and pSp-S, respectively. As shown in Table 1, the comparative EOP analysis revealed broad host ranges for the two phages: phage pSp-J infected 29 (72.5%) MRSP strains and 12 (92%) MSSP strains, while phage pSp-S infected 36 (90%) MRSP strains and 11 (84%) MSSP strains. Having complementary host ranges, our phages could infect all the MSSP strains while they could not infect only three MRSP strains (C28-6-1, Kw34-5-2, and Kw28-5).

### Biological Characteristics of Bacteriophages pSp-J and pSp-S

**Figure 1:**
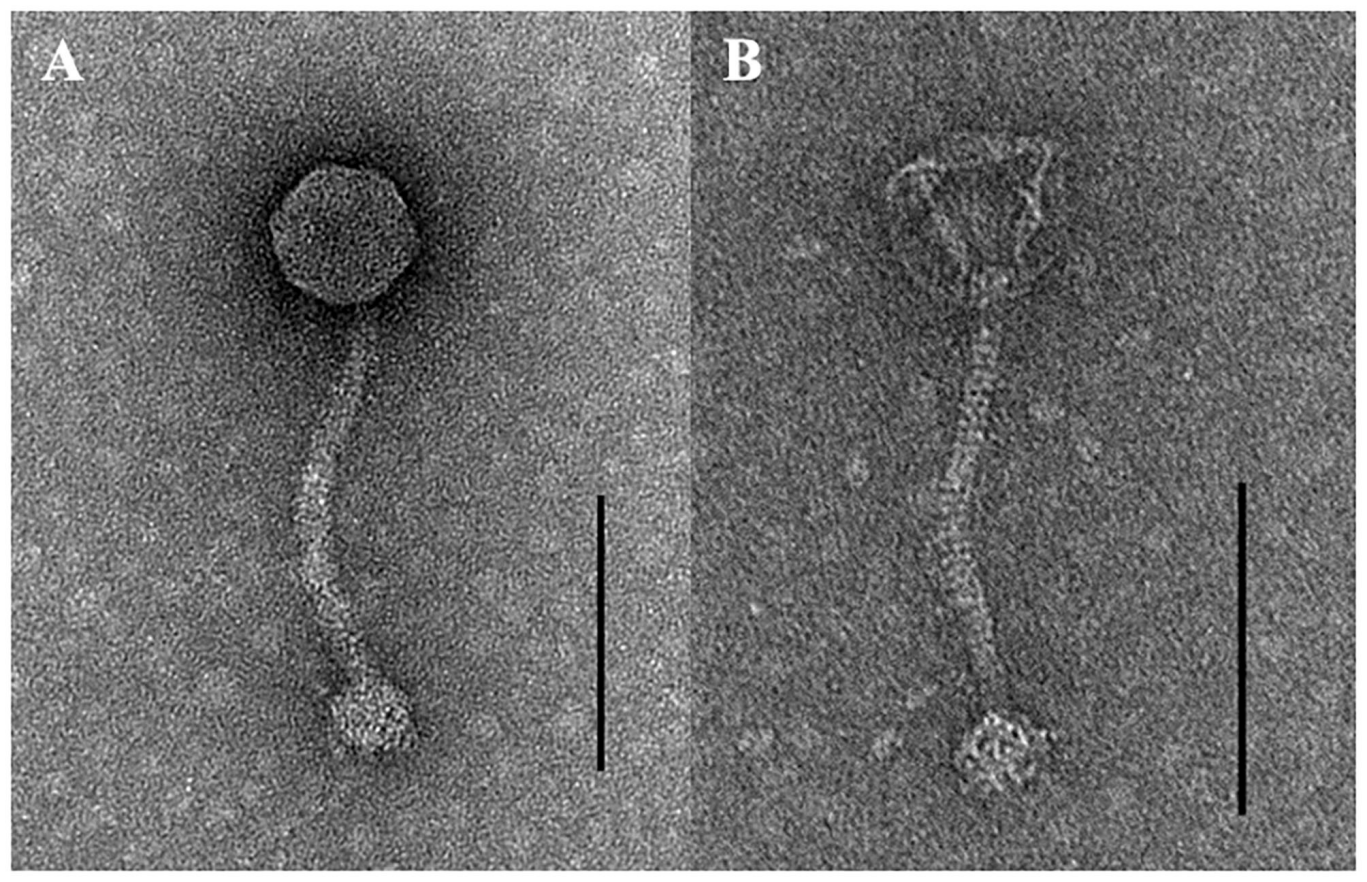
Transmission electron microscopy images of **(A)** pSp-J and **(B)** pSp-S. Scale bar = 100 nm.

**Figure 2:**
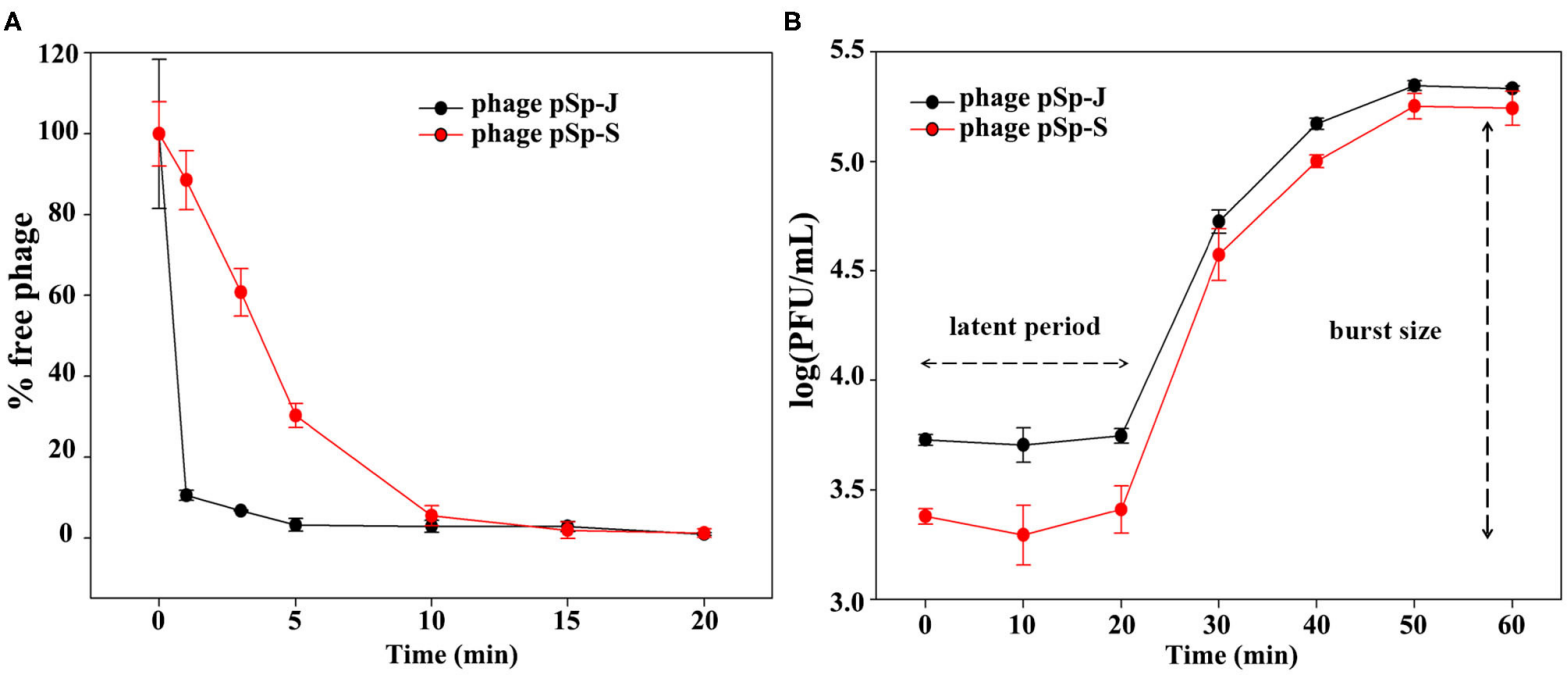
Biological characteristics of pSp-J and pSp-S. **(A)** Adsorption assays and **(B)** one-step growth curve of pSp-J and pSp-S with methicillin resistant *S. pseudintermedius* indicator strain C21-2-1. Values are presented as the mean ± standard deviation.

**Figure 3:**
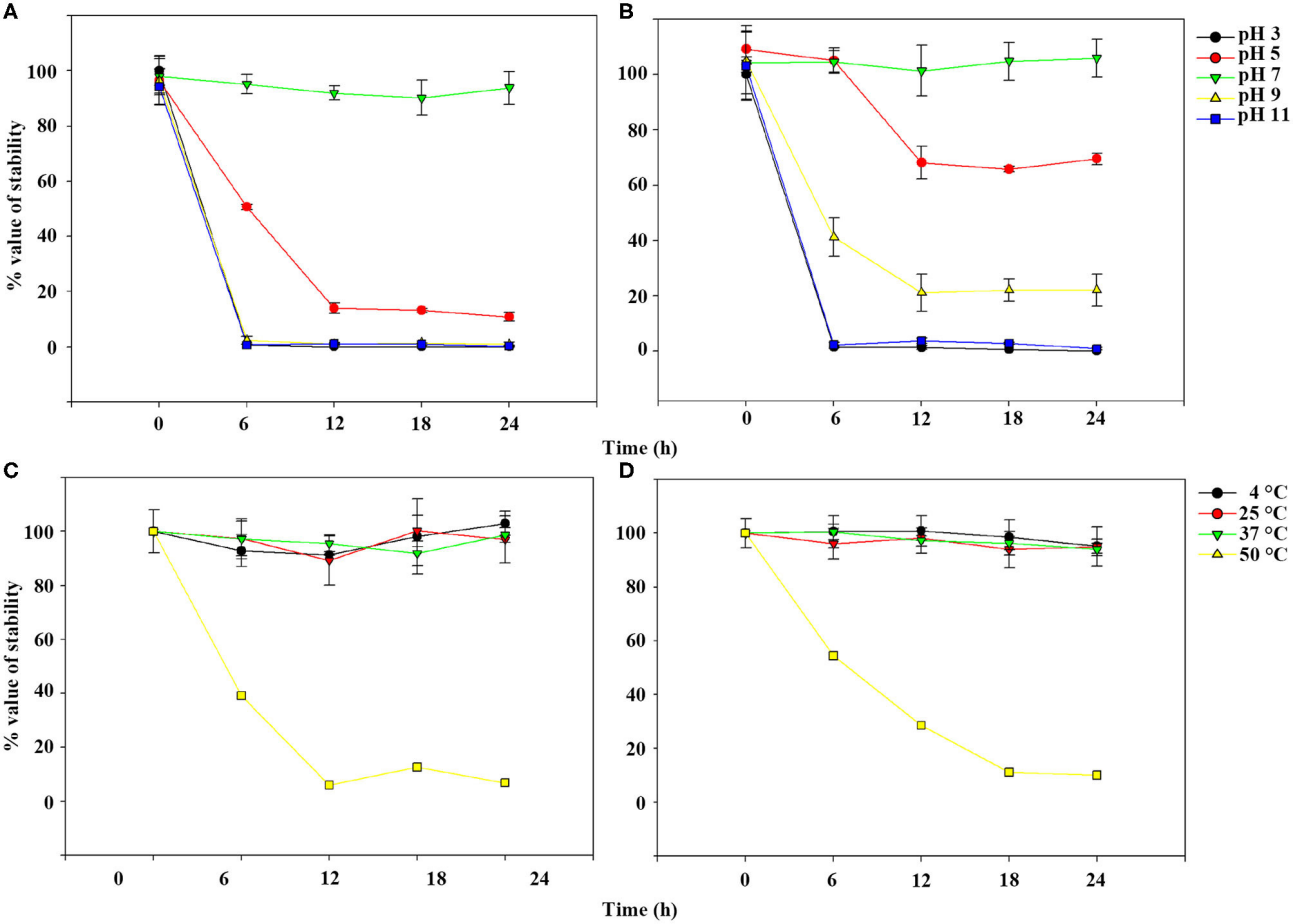
Phage stability of pSp-J and pSp-S at different pH [pSp-J **(A)** and pSp-S **(B)**] and temperature (pSp-J **(C)** and pSp-S **(D)**] conditions. Phages were incubated for 24 h under each condition and the phage titer was determined on the host strain at 6 h intervals. Values are presented as the mean ± standard deviation.

TEM examination revealed that phage pSp-J and pSp-S both have icosahedral heads of 50.96 ± 2.68 and are 51.17 ± 3.14 nm in diameter; they have non-contractile tails around 155.1 ± 7.96 and 166.14 ± 6.63 nm in length, and 9.69 ± 0.15 and 9.77 ± 0.23 nm in width, respectively (Figure 1). Based on morphology, the two phages were assigned to the siphovirus. Phage adsorption assay revealed that ~95% of the pSp-J virions were adsorbed within 5 min, while 70% of the pSp-S virions were adsorbed to *S. pseudintermedius* within 5 min and over 95% of the pSp-S virions were adsorbed within 10 min (Figure 2A). The growth pattern and burst size of pSp-J and -S revealed that the latent period was 20 min for both, and the burst size was 41 and 75 PFU/infected cell, respectively (Figure 2B). The stability of phage particle on pH and temperature was assessed for 24 h (Figure 3). Both phage pSp-J and -S were stable at pH 7, and their lytic activities were compromised by pH 5 (Figures 3A,B). The phages were observed to have reduced stability at pH 3 and 11. Phage pSp-J and -S were stable at 4, 25, and 37°C, however, the phages lost integrity at 50°C (Figures 3C,D); within 6 h, the infectivity of pSp-J and pSp-S had dropped to 39 and 54%, respectively.

### Effects of Phages on Planktonic Bacterial Cell Lysis

**Figure 4:**
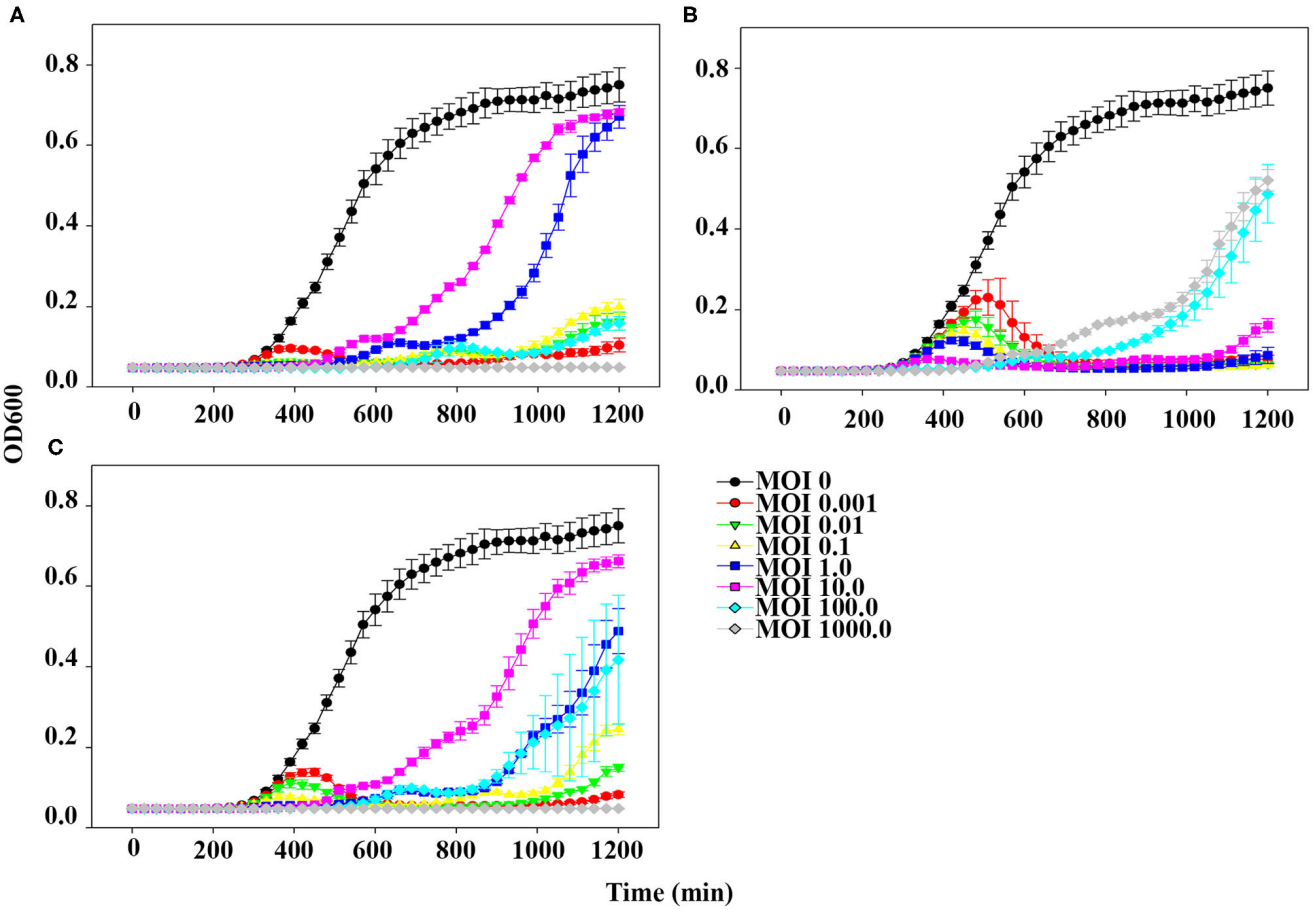
The kinetics for planktonic bacterial cell lysis by **(A)** pSp-J, **(B)** pSp-S, and **(C)** the phage cocktail. Lytic activity was tested on methicillin resistant *S. pseudintermedius* C21-2-1 strain with phages at different concentrations, namely multiplicity of infection (MOI) of 0 (control), 0.001, 0.01, 0.1, 1, 10, 100, and 1000. Values are presented as the mean ± standard deviation.

The planktonic bacterial cell lysis effect was evaluated for 20 h by measuring changes in OD_600_ every 30 min (Figure 4). At the lowest MOI (0.001), host bacteria was able to grow in the presence of phage pSp-J for the first 6 h, after which the growth of the host was inhibited (Figure 4A). However, at higher MOIs (0.01, 0.1, 1, 10, and 100), pSp-J controlled the growth of the host for the first 9 h, following which the host bacteria was able to grow in the MOIs of phage pSp-J. Although the exact MOI values were different, similar trends (i.e., at low MOI the host initially grew but was eventually suppressed, and at high MOI, the host was initially suppressed but eventually grew), were observed for phage pSp-S and the cocktail phage solution experiment, as shown in Figures 4B,C.

### Biofilm Prevention by pSp-J and pSp-S

**Figure 5:**
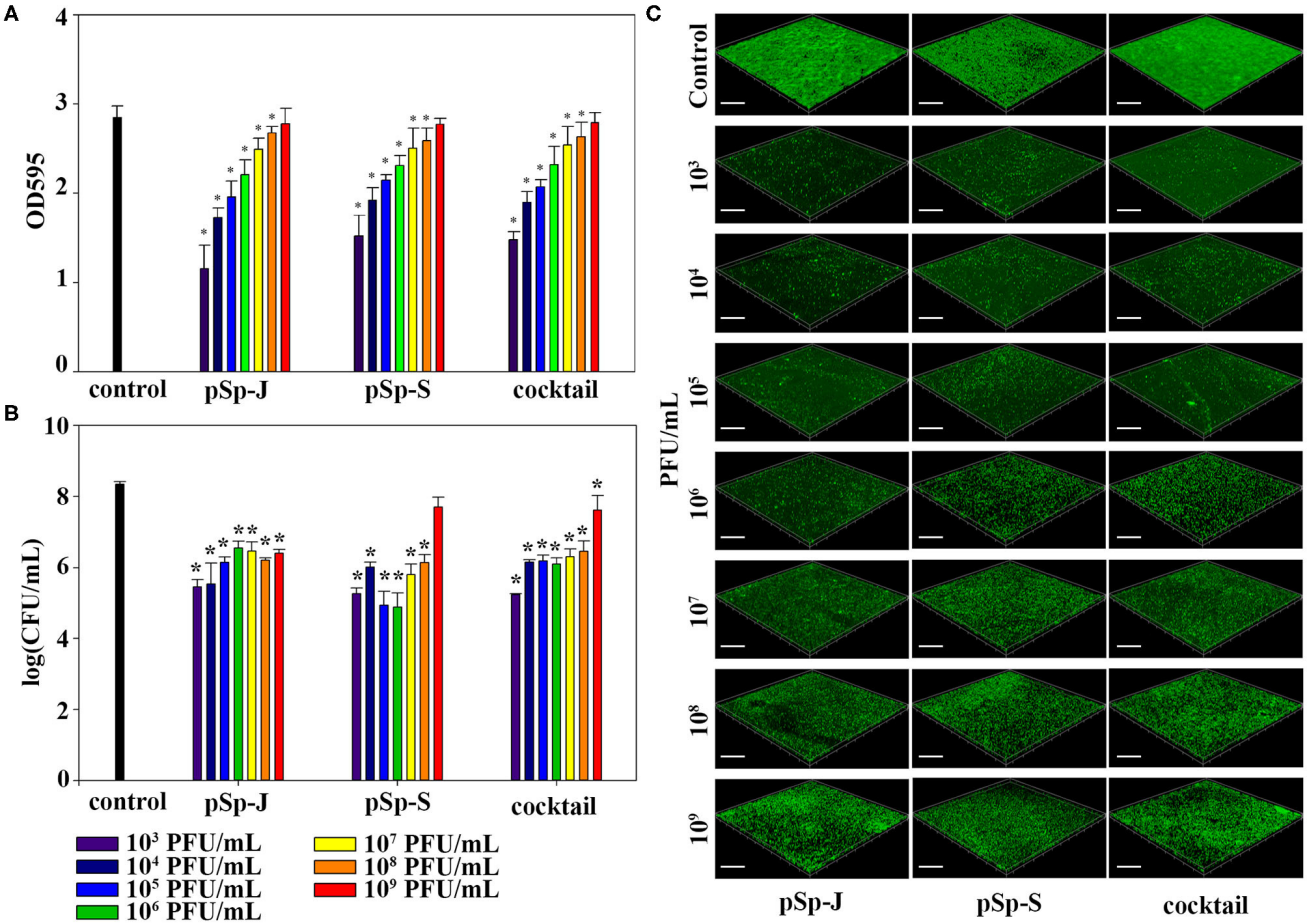
The endpoint reaction measurement for biofilm prevention by pSp-J, pSp-S, and the phage cocktail. **(A)** The total biomass of the biofilm was stained with crystal violet and the optical density (OD) at 595 nm was measured. **(B)** Viable bacterial cell count in the biofilm was measured by the standard plate count and **(C)** visualized with a confocal laser scanning microscope. Scale bar = 100 μm. Values are presented as the mean ± standard deviation; a one-way ANOVA (Bonferroni *post-hoc* test) was performed to determine statistically significant differences (*P* < 0.05) between groups. Asterisk indicates statistical significance between the experimental and control group.

To evaluate the ability of phages to prevent biofilm formation, different concentrations of phages (pSp-J, pSp-S, or the phage cocktail) were co-cultured with the host bacteria for 24 h without shaking, and changes in the degree of staining with crystal violet and changes in CFU were measured for total biomass and viable bacterial load, respectively. As shown in Figure 5A, both the phage pSp-J and pSp-S showed significant effect (*P* < 0.05) in prevent total biomass of the biofilm (Figure 5A) at their low concentrations (10^3^, 10^4^, 10^5^, 10^6^, 10^7^, and 10^8^ PFU/mL treatment). The host cell viability in the biofilms was effectively reduced by lower concentrations for pSp-J, pSp-S, and phage cocktail (Figure 5B). The biofilm prevention efficacy of phages was visualized with CLSM (Figure 5C). The pSp-J, pSp-S, and phage cocktail prevented the formation of biofilm by inhibiting the growth of viable bacterial cells with an inverse concentration-dependent manner.

### Biofilm Degradation by pSp-J and pSp-S

**Figure 6:**
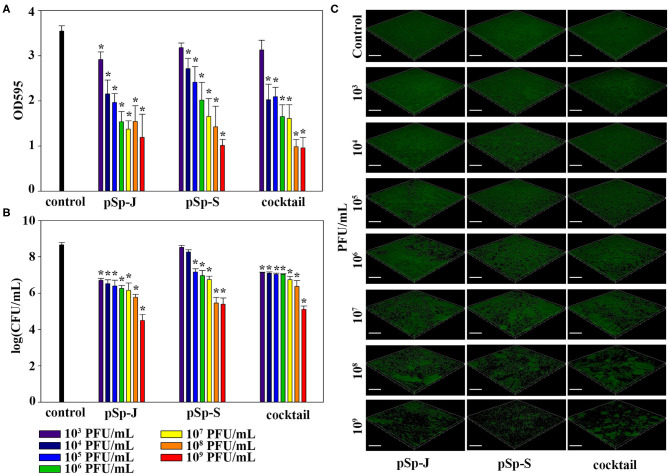
The endpoint reaction measurement for biofilm degradation by pSp-J, pSp-S, and the phage cocktail. **(A)** The total biomass of biofilm was stained with crystal violet and the optical density (OD) at 595 nm was measured. **(B)** The viable bacterial cell count in the biofilm was measured by the standard plate count and **(C)** visualized with a confocal laser scanning microscope. Scale bar = 100 μm. Values are presented as the mean ± standard deviation, and a one-way ANOVA (Bonferroni *post-hoc* test) was performed to determine statistically significant differences (*P* < 0.05) between groups. Asterisk indicates statistical significance between the experimental and control group.

To evaluate the ability of phages to degrade biofilm, different concentrations of phages (pSp-J, pSp-S, or the phage cocktail) were used to treat 24 h-old *S. pseudintermedius* biofilm for 24 h without shaking. The changes in the intensity of the crystal violet stain and in CFU were measured for total biomass and viable bacterial load, respectively. The total biomass of the biofilm decreased in a concentration-dependent manner and significantly decreased at 10^3^-10^9^ PFU/ml of pSp-J (*P* < 0.05), at 10^4^-10^9^ PFU/mL of pSp-S (*P* < 0.05), and at 10^4^-10^9^ PFU/ml of phage cocktail (*P* < 0.05; Figure 6A). Concentration-dependent biofilm degradation was also confirmed by the viable bacterial cell count in the biofilms (Figure 6B). The ability of phages to degrade biofilm was visualized through CLSM (Figure 6C). The pSp-J, pSp-S, and phage cocktail lysed the viable bacterial cells in biofilm in a concentration-dependent manner.

### Bioinformatic Analysis of pSp-J and pSp-S

**Figure 7:**
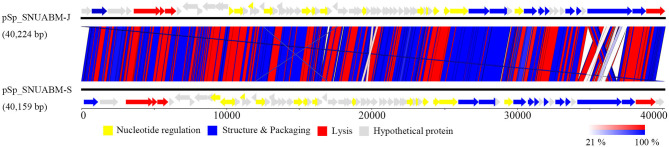
The whole genome map of the phage pSp-J, and pSp-S. Predicted ORFs are classified according to their function; yellow represents nucleotide metabolism related proteins, blue represents structural and packaging related proteins, red represents lysis related proteins, and gray represents hypothetical proteins. (Scale = base pairs).

**Figure 8:**
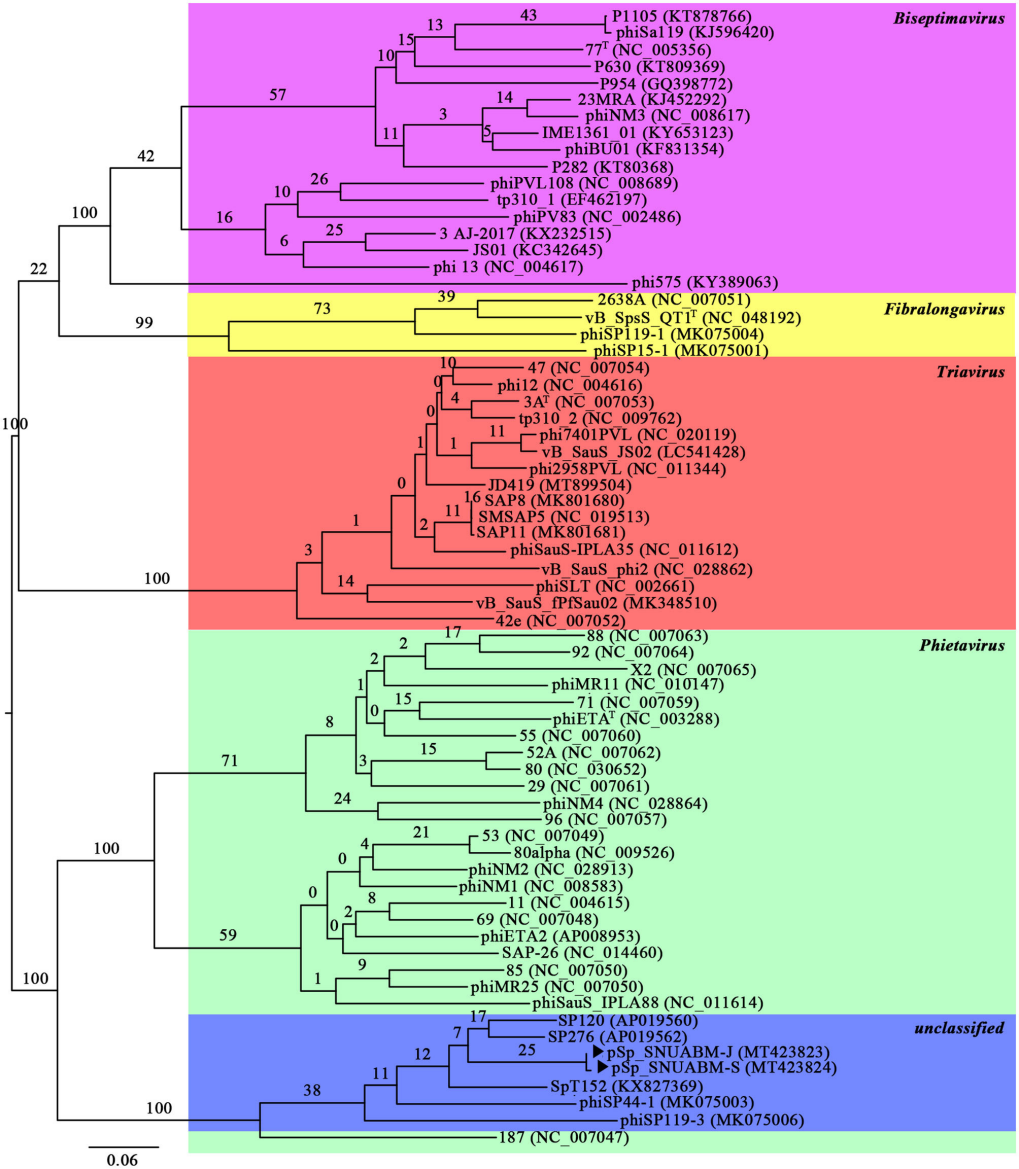
The phylogenetic analysis of the phage pSp-J, and pSp-S. The tree was constructed using VICTOR with recommended settings for phages (prokaryotic viruses). The clusters of phages are colored in green (*Phietavirus*), blue (unclassified), purple (*Biseptimavirus*), yellow (*Fibralongavirus*), and red (*Triavirus*).

A total of 10,298,520 reads (1,554,925,520 bp) were obtained for pSp-J with average genome coverage of 380. The complete genome of pSp-J (40,224 bp) contained 74 ORFs which are majority found on the positive strand (66 ORFs). A total of 10,383,812 reads (1,567,955,612 bp) were obtained for pSp-S with average genome coverage of 381. The complete genome of pSp-S (40,159 bp) contained 72 ORFs which also are majorly found on the positive strand (64 ORFs). The tRNA, antimicrobial resistance-, or virulence- related genes was not detected in both phages. The predicted function of the ORFs was classified into the following four categories: nucleotide metabolism (e.g., HNHc nuclease, single-stranded DNA binding protein), structure and packaging (e.g., major capsid protein, head-tail connector protein, and terminase large/small subunit), lysis (e.g., holin, lysin, β-N-acetylglucosaminidase), and unknown function (Figure 7). The ORFs of both phages were similarly arranged except pSp-J contained two more hypothetical proteins such as ORF4 (3,106–3,507 bases) and ORF44 (21,158–21,328 bases). Especially, the ORFs associated with structure and packaging were clustered in the last part of the genome of both phages. The features of the predicted ORFs identified in phage pSp-J and -S were listed in Supplementary Tables 2, 3. A nucleotide BLAST result revealed that phage pSp-J and -S have best match with *Staphylococcus* phage 187 (89% identity, and 53% coverage) which is a distinct member of the family *Phietavirus*, a well-known bacteriophage genus included in the *Siphoviridae*, infecting *Staphylococcus* spp. The phylogenetic analysis also showed that the phages isolated in this study constructed cluster with several unclassified siphoviruses and phage 187 in the *Phietavirus* consist of several subgroups rather than any other siphoviruses infecting *Staphylococcus* such as *Biseptimavirus, Fibralongavirus*, and *Triavirus*, which is a morphologically similar genus with *Phietavirus* (Figure 8).

### Pseudolysogeny Analysis

Totally, five colonies of original host and the re-grown bacteria after the treatment of each phages were analyzed. All the assay with chromosomal DNA showed negative for major capsid protein of pSp-J, and pSp-S. We could observe the major capsid protein band (~378 bp) only in the extrachromosomal DNA from the re-grown colonies.

## Discussion

As a zoonotic pathogen, dog to human transmission and implantable material related surgical site infection of *S. pseudintermedius* has been reported in human cases (2, 40, 41). It is well-known that the bacterial biofilms complicate the implant material related surgical site infection (42). In this study, we screened the antibiotic-resistance profiles of 54 strains of *S. pseudintermedius* including 25 strains (including six strains from veterinary staff; isolated in 2012 and 2016) from previous study and newly isolated 29 canine strains (isolated in 2018 and 2019) from this study. The isolated bacteria were highly resistant to methicillin (82.7%), whereas previous studies have reported 40–70% MRSP isolation rates in Korea (39, 43, 44). Multidrug-resistant (MDR) bacteria is a growing concern worldwide, and almost all the MRSP strains in our study were MDR strains. There is currently no official antibiotic stewardship program for the veterinary sector in Korea; thus, the circulation of MDR MRSP among companion canines and their owners could pose a potential problem for the effectivity of antibiotic medication. Therefore, an alternative to antibiotics is needed, and we suggest that bacteriophages be used as biocontrol agents against MDR MRSP.

*S. pseudintermedius* can be isolated from any tissue; however, it is primarily associated with canine pyoderma and otitis externa (39, 45). Thus, for therapeutic applications, the phages have to be stable on the target environment. The isolated phages, pSp-J and pSp-S, showed good stability under thermal or pH stress over the tested periods, and showed potential for use under normal environment of the body such as canine skin or human blood conditions (i.e., ~37°C and pH ~7).

The host range and lytic potential of candidates for phage therapy are also major considerations. The phages specific to *S. pseudintermedius* from a recent study can lyse 100% of the tested MRSP (*n* = 17) strains and 16-28% of the tested MSSP (*n* = 43) strains, depending on the phages (46). In our study, two phages (pSp-J and pSp-S) have shown lytic activity on 68 and 85% of the tested MRSP strains and 92 and 84% of the tested MSSP strains, respectively. The two phages have complementary host ranges, and only three MRSP strains could not be lysed by our phages. Furthermore, the phages could infect the strains isolated from veterinary staff whether it is methicillin resistant strain or not (Table 1).

Interestingly, the lytic kinetics of the phages were far different compared with the trend generally observed for other phages (i.e., most phages demonstrate concentration-dependent lytic activity) (47, 48). In our study, upon treatment with low concentrations of phages, bacteria initially grew for several hours, after which bacterial growth was inhibited. In contrast, upon treatment with high concentrations of phages, bacterial growth was inhibited during the first few hours, after which the bacteria began to grow (Figure 4). This peculiar anti-bacterial effect of phages has been observed in various environments and can be attributed to pseudolysogeny induced by a high MOI phage (49). Pseudolysogeny of virulent phages is considered to provide advantages such as long term survival of their genome in the condition for propagation is not favorable (50, 51). This evolutionary characteristic of the pseudolysogeny is a disadvantage for phage therapy with the following flaws: hindrance of other phage-pathogen collision by viscous materials originated from host cell lysis; transduction of antibiotic/phage resistant-, virulence-related genes (49). As a high dose of phage solution is preferable for therapeutic application, it seems like a negative effect that re-growth of the pathogen is observed as a result of pseudolysogeny induction in high MOI groups. And the phages having pseudolysogeny life cycle are not recommended for the therapeutic agent (49, 50). However, the antimicrobial effect of two newly isolated phages was superior to the high MOI groups at their low MOI (Figure 4), which can be an economical way to treat the pathogen in case the induction of pseudolysogeny can be prevented. Good examples to overcome the pseudolysogeny phages have been reported (52, 53).

For biofilm-associated infection, antibiotics are not the perfect choice since biofilm formation correlates with high antibiotic tolerance (11). Previous studies have reported that phages and their derivatives, such as endolysin, have anti-biofilm potential against staphylococci (13, 54, 55). As we have observed in the planktonic cell lysis assay, biofilm formation was inhibited by low concentrations of phages (Figure 5). The Anti-biofilm effect of the phages may also be considered to be concentration-dependent as revealed in the previous studies (56–58). However, too high concentration of phages can interfere with the disruption of biofilm, and too low concentration of phages may not be sufficient to infect and penetrate the biofilm (59). Treatment time may be a major factor for the biofilm disruption rather than the concentration (14). Thus, we suggest that the appropriate concentration of the phages should be applied according to the usage.

Although the phages have been suggested as potential alternatives to antibiotics, bacterial resistance to phages is a well-known phenomenon. The application of different phages in a combination, or a cocktail of phages, can be used to overcome this. Phage cocktails bypass the resistance to a single phage, broaden the treatment choice against multiple pathogens, and take advantage of the phages' synergistic effects (60–62). However, in our study, synergy between the two phages was not observed against both planktonic and biofilm bacterial cells. However, the two phages have different host ranges and thus can complement each other to broaden bacterial host coverage.

One of the major concerns on phage therapy is the possibility of transmission the antimicrobial-resistant- or virulence-related genes (63, 64). However, those harmful genes were not detected in the genomes of pSp-J and pSp-S (Figure 7 and Supplementary Tables 2, 3). Based on the terminase homology, the two phages having virulent life cycle considered to have a headful packaging strategy, which can induce accidental generalized transduction. Although the generalized transduction frequency can be low as 10^−11^ PFU, a large number of phages used for the biocontrol agent may increase the likelihood of generalized transduction (65). In addition, our study revealed that higher phage concentrations could induce re-growth of the pathogen by pseudolysogeny, in the end. In summary, we suggest if pseudolysogenic phages should be used a relatively low dose of phages can be more effective in therapeutic purposes and minimize the transduction of harmful genes.

Overall, we have successfully isolated two bacteriophages, pSp-J and pSp-S, that infect *S. pseudintermedius*, and we were able to characterize these phages for application on biofilm prevention and degradation. Our results revealed the potential of these bacteriophages and their cocktail as alternatives to antibiotics in treating *S. pseudintermedius*. Further studies on the mechanism behind the poor bacterial growth inhibition capacities of high concentrations of phages can be performed to help us understand and maximize the application of these phages in treating *S. pseudintermedius*.

## Data Availability Statement

All datasets generated for this study are included in the article/Supplementary Material.

## Author Contributions

SGK and SP contributed conceptualization and design of the study. SGK, SWK, SH, and WO conducted the experiments. SGK, SG, SY, SL, and JK analyzed and validate the data. SGK, YP, and SP supervised and reviewed the manuscript. All authors contributed to the article and approved the submitted version.

## Conflict of Interest

The authors declare that the research was conducted in the absence of any commercial or financial relationships that could be construed as a potential conflict of interest.
