# Supplementary material for: Two Novel Bacteriophages Control Multidrug- and Methicillin-Resistant Staphylococcus pseudintermedius Biofilm
Source: Front Med (Lausanne). 2021 Mar 31;8:524059. doi: 10.3389/fmed.2021.524059 (PMC8044756; doi:10.3389/fmed.2021.524059)
Supplement: Supplementary file 1 [file Table_1.docx]

**Table S1.** Antimicrobial susceptible profiles of *S. pseudintermedius* from canine origin.

| **MR status** | **Strain** | **Antimicrobial agents** | | | | | | | | | | | | | | | **Source** |
| --- | --- | --- | --- | --- | --- | --- | --- | --- | --- | --- | --- | --- | --- | --- | --- | --- | --- |
|  |  | **P** | **OXA** | **GEN** | **AN** | **SYN** | **RA** | **CHL** | **SXT** | **CIP** | **LEV** | **MI** | **TE** | **LZD** | **E** | **CLI** |  |
| MR^a^ | 1D1 | R^b^ | R |  |  |  |  | R | R | R | R |  | R |  | R | R | [22] |
|  | 1D1V | R | R |  |  |  |  | R | R | R | R |  | R |  | R | R |  |
|  | 3H1-2 | R | R | R |  |  |  | R | R | R |  |  | R |  | R | R |  |
|  | 3H1-2V | R | R | R |  |  |  | R | R |  |  |  | R |  | R | R |  |
|  | 4D1 | R | R |  |  |  |  |  | R |  |  | R | R |  |  |  |  |
|  | 4D1V | R | R |  |  |  |  |  | R |  |  |  | R |  |  |  |  |
|  | 5D1 | R | R | R |  |  |  |  | R |  |  |  | R |  |  |  |  |
|  | 5D1V | R | R | R |  |  |  |  | R |  |  |  | R |  |  |  |  |
|  | 7D1 | R | R | R |  |  |  | R | R | R | R |  | R |  | R | R |  |
|  | 8D1V | R | R | R |  |  |  |  | R | R | R |  | R |  | R | R |  |
|  | 8H1-10 |  | R |  |  |  |  |  |  |  |  |  |  |  |  |  |  |
|  | 9D1 | R | R |  |  |  |  | R | R | R | R |  | R |  | R |  |  |
|  | 9D1V | R | R | R |  |  |  | R | R | R | R |  | R |  | R |  |  |
|  | 32-2 | R | R |  |  |  |  |  | R | R | R |  | R |  | R | R |  |
|  | 47-5 | R | R |  |  |  |  |  | R | R | R |  | R |  | R | R |  |
|  | 54-1 | R | R |  |  |  |  |  | R | R | R |  | R |  | R | R |  |
|  | A-ISB | R | R |  |  |  |  |  | R | R | R |  | R |  | R | R |  |
|  | C21-2-1 | R | R |  |  |  |  | R |  |  |  |  |  |  |  |  | This study |
|  | C27-6 | R | R |  |  |  |  |  |  | R | R |  | R |  |  |  |  |
|  | C28-6-1 | R | R |  |  |  |  |  | R | R | R |  | R |  |  |  |  |
|  | C28-6-2 | R | R |  |  |  |  | R | R | R | R |  | R |  | R | R |  |
|  | C30-4-1 | R | R |  |  |  |  |  | R | R | R |  | R |  |  |  |  |
|  | C40-4-1 | R | R |  |  |  |  | R | R |  |  |  | R |  | R |  |  |
|  | C41-4 | R | R |  |  |  |  | R | R |  |  |  | R |  | R | R |  |
|  | C49-1 | R | R | R |  |  |  |  | R | R | R | R | R |  | R | R |  |
|  | C55-4-1 | R | R |  |  |  |  | R | R |  |  |  | R |  | R | R |  |
|  | C56-4-1 | R | R |  |  |  |  | R | R |  |  |  | R |  | R | R |  |
|  | J37-2 | R | R |  |  |  |  |  |  |  |  |  | R |  |  |  |  |
|  | Kw34-4-2 | R | R |  |  |  |  | R | R |  |  |  | R |  | R | R |  |
|  | Kw34-3 | R | R | R |  |  |  | R | R |  |  |  | R |  | R | R |  |
|  | Kw34-5-2 | R | R |  |  |  |  | R | R |  |  |  | R |  | R | R |  |
|  | Kw28-1 | R | R | R |  |  |  | R | R | R | R |  | R |  |  |  |  |
|  | Kw28-5 | R | R |  |  |  |  | R | R | R | R |  | R |  |  |  |  |
|  | Kw33-3-2 | R | R |  |  |  |  |  |  |  |  |  |  |  |  |  |  |
|  | Kw34-2 | R | R |  |  |  |  | R | R |  |  |  | R |  | R | R |  |
|  | In36-4 | R | R |  |  |  |  | R | R |  |  |  | R |  | R | R |  |
|  | In37-4-1 |  | R |  |  |  |  |  |  |  |  |  | R |  |  |  |  |
|  | In47-4-1 | R | R | R |  |  |  |  | R |  |  |  | R |  |  |  |  |
|  | In47-4-3 | R | R |  |  |  |  |  | R |  |  |  | R |  |  |  |  |
|  | In48-4-2 | R | R |  |  |  |  |  | R |  |  |  | R |  |  |  |  |
|  | In50-4-1 | R | R | R |  |  |  | R | R |  |  |  | R |  | R | R |  |
| MS^a^ | 6D1 | R |  |  |  |  |  |  | R | R | R |  | R |  | R | R | [22] |
|  | 6D1V | R |  |  |  |  |  |  | R | R | R |  |  |  | R | R |  |
|  | 8D1 | R |  | R |  |  |  |  | R | R | R |  | R |  | R | R |  |
|  | 8H1-10V |  |  |  |  |  |  |  |  |  |  |  |  |  |  |  |  |
|  | D10-3 | R |  |  |  |  |  | R | R |  |  |  | R |  | R |  |  |
|  | D13-1 | R |  | R |  |  |  |  | R | R | R |  | R |  | R | R |  |
|  | D20 | R |  | R |  |  |  | R | R |  |  |  | R |  | R | R |  |
|  | A-D4-1 | R |  | R |  |  |  |  | R | R | R |  | R |  | R | R |  |
|  | Kw31-4-3 | R |  |  |  |  |  |  |  |  |  | R | R |  |  |  | This study |
|  | Kw41-4-1 | R |  |  |  |  | R | R | R |  |  |  |  |  | R | R |  |
|  | In41-4-2 | R |  |  |  |  |  | R | R |  |  |  |  |  | R | R |  |
|  | In42-4-2 | R |  |  |  |  |  |  |  |  |  |  |  |  |  |  |  |
|  | In55-4-3 |  |  |  |  |  |  | R | R |  |  |  |  |  | R | R |  |

^a^ MR, methicillin-resistance; MS, methicillin-susceptible.

^b^ R, resistance

**Table S2** Functional categories of the predicted genes in *S. pseudintermedius* phage pSp-J.

| **Group** | **Locus tag** | **Encoded protein** | **Related organism** | **Query coverage (%)** | **Identity**  **(%)** |
| --- | --- | --- | --- | --- | --- |
| Structure and packaging | pSpJ_02 | Putative minor structural protein | *Staphylococcus* phage SP120 | 99 | 97.12 |
| Nucleotide regulation | pSpJ_05 | Putative Beta-N-acetylglucosaminidase | *Staphylococcus* phage phiSP119-3 | 99 | 98.73 |
| Lysis | pSpJ_06 | Putative holin | *Staphylococcus* phage phiSp44-1 | 98 | 97.62 |
| Lysis | pSpJ_07 | Putative lysin | *Staphylococcus* phage phiSP119-3 | 99 | 96.81 |
| Nucleotide regulation | pSpJ_09 | Putative integrase | *Staphyloccocus* | 99 | 100 |
| Nucleotide regulation | pSpJ_14 | Putative DNA binding protein | *Staphylococcus* phage phiSP119-1 | 75 | 99.07 |
| Nucleotide regulation | pSpJ_15 | Putative antirepressor protein | *Staphylococcus* pahge phiSP119-1 | 99 | 86.4 |
| Nucleotide regulation | pSpJ_17 | Putative CRISPR associated protein | *Staphylococcus* phage SpT152 | 98 | 98.85 |
| Nucleotide regulation | pSpJ_21 | Putative antirepressor protein | *Staphylococcus* phage IME1365_01 | 99 | 90.62 |
| Nucleotide regulation | pSpJ_29 | Putative single-stranded DNA-binding protein | *Staphylococcus* phage SP120 | 99 | 99.55 |
| Nucleotide regulation | pSpJ_30 | Putative single-stranded DNA-binding protein | *Staphylococcus* phage SpT152 | 99 | 96.55 |
| Nucleotide regulation | pSpJ_32 | Putative HNHc nuclease | *Staphylococcus* phage SpT152 | 99 | 94.22 |
| Nucleotide regulation | pSpJ_35 | helix turn helix domain containing protein | *Staphylococcus* phage SpT99F3 | 99 | 99.16 |
| Nucleotide regulation | pSpJ_38 | Putative endodeoxyribonulcease | *Staphylococcus* phage phiSP44-1 | 96 | 98.54 |
| Nucleotide regulation | pSpJ_52 | Putative dUTPase | *Staphylococcus* phage SP197 | 99 | 89.89 |
| Nucleotide regulation | pSpJ_54 | Putative transcriptional activator (RinB like) | *Staphylococcus* phage phiSP38-1 | 96 | 60 |
| Nucleotide regulation | pSpJ_56 | Putative RNA polymerase sigma 70 factor | *Staphylococcus* phage phi575 | 92 | 43.18 |
| Structure and packaging | pSpJ_57 | Putative terminase small subunit | *Staphylococcus* phage Sp120 | 99 | 97.28 |
| Structure and packaging | pSpJ_58 | Putative terminase large subunit | *Staphylococcus* virus 187 | 99 | 92.92 |
| Structure and packaging | pSpJ_59 | Putative portal protein | *Staphylococcus* phage SP120 | 99 | 98.54 |
| Structure and packaging | pSpJ_60 | Putative capsid morphogenesis protein (Ntox50 and MuF domain containing protein) | *Staphylococcus* phage SP276 | 98 | 94.13 |
| Nucleotide regulation | pSpJ_62 | PRK00409 domain containing protein | *Staphylococcus* phage SP276 | 99 | 96.55 |
| Structure and packaging | pSpJ_63 | Putative major capsid protein | *Staphylococcus* phage phiSP44-1 | 99 | 97.69 |
| Structure and packaging | pSpJ_64 | Putative major tail protein | *Staphylococcus* phage phiSP44-1 | 99 | 97.32 |
| Structure and packaging | pSpJ_65 | Putative head-tail connector protein | *Staphylococcus* virus 187 | 99 | 97.27 |
| Structure and packaging | pSpJ_69 | Putative major tail protein | *Staphylococcus* phage SP120 | 99 | 96.77 |
| Structure and packaging | pSpJ_70 | Putative tail assembly chaperone protein | *Staphylococcus* phage StB12 | 84 | 52.78 |
| Structure and packaging | pSpJ_72 | Putative tail length measure protein | *Staphylococcus* phage SpT152 | 99 | 92.51 |
| Structure and packaging | pSpJ_73 | Putative tail family protein | *Staphylococcus* virus 187 | 99 | 93.23 |
| Lysis | pSpJ_74 | Putative tail endopeptidase | *Staphylococcus* phage SpT152 | 99 | 99.38 |

**Table S3** Functional categories of the predicted genes in *S. pseudintermedius* phage pSp-S.

| **Group** | **Locus tag** | **Encoded protein** | **Related organism** | **Query coverage (%)** | **Identity**  **(%)** |
| --- | --- | --- | --- | --- | --- |
| Structure and packaging | pSpS_01 | Putative minor structural protein | *Staphylococcus* phage SP120 | 97 | 97.55 |
| Nucleotide regulation | pSpS_03 | Putative beta-N-acetylglucosaminidase | *Staphylococcus* phage phiSP119-3 | 99 | 98.73 |
| Lysis | pSpS_04 | Putative holin | *Staphylococcus* phage phiSp44-1 | 98 | 97.62 |
| Lysis | pSpS_05 | Putative lysin | *Staphylococcus* phage phiSP119-3 | 99 | 96.81 |
| Nucleotide regulation | pSpS_7 | Putative integrase | *Staphylococcus* | 99 | 100 |
| Nucleotide regulation | pSpS_11 | Putative repressor | *Staphylococcus* phage phiSP119-3 | 95 | 74.15 |
| Nucleotide regulation | pSpS_12 | Putative DNA binding protein | *Staphylococcus* phage phiSP119-1 | 75 | 99.07 |
| Nucleotide regulation | pSpS_13 | Putative antirepressor | *Staphylococcus* phage SpT119-1 | 99 | 86.4 |
| Nucleotide regulation | pSpS_15 | Putative CRISPR associated protein | *Staphylococcus* phage SpT152 | 98 | 98.85 |
| Nucleotide regulation | pSpS_19 | Putative antirepressor and regulatory protein Rha | *Staphylococcus* phage IME1365_01 | 99 | 90.62 |
| Nucleotide regulation | pSpS_27 | Putative single-stranded DNA binding protein | *Staphylococcus* phage SP120 | 99 | 99.55 |
| Nucleotide regulation | pSpS_28 | Putative single-stranded DNA binding protein | *Staphylococcus* phage SP197 | 99 | 84.14 |
| Nucleotide regulation | pSpS_30 | Putative HNHc nuclease | *Staphylococcus* virus X2 | 97 | 91.28 |
| Nucleotide regulation | pSpS_33 | helix-turn-helix domain containing protein | *Staphylococcus* phage SpT99F3 | 99 | 99.16 |
| Nucleotide regulation | pSpS_34 | AAA domain containing protein | *Staphylococcus* virus 187 | 97 | 84.27 |
| Nucleotide regulation | pSpS_36 | Putative endodeoxyribonuclease | *Staphylococcus* phage phiSP44-1 | 96 | 98.54 |
| Nucleotide regulation | pSpS_49 | Putative dUTP pyrophosphatase | *Staphylococcus* phage SP197 | 99 | 89.89 |
| Nucleotide regulation | pSpS_51 | Putative transcriptional activator (RinB like) | *Staphylococcus* phage phiSP38-1 | 96 | 60 |
| Nucleotide regulation | pSpS_53 | Putative RNA polymerase sigma 70 factor | *Staphylococcus* phage phi575 | 92 | 43.18 |
| Structure and packaging | pSpS_54 | Putative terminase small subunit | *Staphylococcus* phage SP120 | 99 | 97.28 |
| Structure and packaging | pSpS_55 | Putative terminase large subunit | *Staphylococcus* phage SP187 | 99 | 92.92 |
| Structure and packaging | pSpS_56 | Putative portal protein | *Staphylococcus* phage SP120 | 99 | 98.54 |
| Structure and packaging | pSpS_57 | Putative capsid morphogenesis protein (Ntox50 and MuF domain containing protein) | *Staphylococcus* phage SP276 | 98 | 94.13 |
| Nucleotide regulation | pSpS_59 | PRK00409 domain containing protein | *Staphylococcus* phage SP276 | 99 | 90.64 |
| Structure and packaging | pSpS_60 | Putative major capsid protein | *Staphylococcus* phage phiSP44-1 | 99 | 89.77 |
| Structure and packaging | pSpS_61 | Putative major tail protein | *Staphylococcus* phage phiSP44-1 | 99 | 96.64 |
| Structure and packaging | pSpS_62 | Putative head tail connector protein | *Staphylococcus* virus 187 | 99 | 97.27 |
| Structure and packaging | pSpS_64 | HK97 gp10 domain containing protein | *Staphylococcus* phage SP120 | 99 | 65.31 |
| Structure and packaging | pSpS_66 | Putative major tail protein | *Staphylococcus* phage SP120 | 99 | 96.77 |
| Structure and packaging | pSpS_67 | Putative tail assembly chaperone protein | *Staphylococcus* phage StB12 | 84 | 52.78 |
| Structure and packaging | pSpS_69 | Putative tail length tape-measure protein | *Staphylococcus* phage SP276 | 84 | 94.99 |
| Structure and packaging | pSpS_70 | Putative tail family protein | *Staphylococcus* virus 187 | 99 | 93.23 |
| Lysis | pSpS_71 | Putative tail endopeptidase | *Staphylococcus* phage SpT152 | 99 | 98.49 |
